# Machine learning-informed predictions of nanoparticle mobility and fate in the mucus barrier

**DOI:** 10.1063/5.0091025

**Published:** 2022-06-21

**Authors:** Logan Kaler, Katherine Joyner, Gregg A. Duncan

**Affiliations:** 1 Biophysics Program, University of Maryland, College Park, Maryland 20742, USA; 2 Fischell Department of Bioengineering, University of Maryland, College Park, Maryland 20742, USA

## Abstract

Nanomaterial diffusion through mucus is important to basic and applied areas of research such as drug delivery. However, it is often challenging to interpret nanoparticle dynamics within the mucus gel due to its heterogeneous microstructure and biochemistry. In this study, we measured the diffusion of polyethylene glycolylated nanoparticles (NPs) in human airway mucus *ex vivo* using multiple particle tracking and utilized machine learning to classify diffusive vs sub-diffusive NP movement. Using mathematic models that account for the mode of NP diffusion, we calculate the percentage of NPs that would cross the mucus barrier over time in airway mucus with varied total solids concentration. From this analysis, we predict rapidly diffusing NPs will cross the mucus barrier in a physiological timespan. Although less efficient, sub-diffusive “hopping” motion, a characteristic of a continuous time random walk, may also enable NPs to cross the mucus barrier. However, NPs exhibiting fractional Brownian sub-diffusion would be rapidly removed from the airways via mucociliary clearance. In samples with increased solids concentration (>5% w/v), we predict up to threefold reductions in the number of nanoparticles capable of crossing the mucus barrier. We also apply this approach to explore diffusion and to predict the fate of influenza A virus within human mucus. We predict only a small fraction of influenza virions will cross the mucus barrier presumably due to physical obstruction and adhesive interactions with mucin-associated glycans. These results provide new tools to evaluate the extent of synthetic and viral nanoparticle penetration through mucus in the lung and other tissues.

## I. INTRODUCTION

Airway mucus acts as a natural filter to capture inhaled particulates by coating lung epithelial surfaces and is a critical component of innate defenses in the respiratory tract. Mucus is comprised of secreted gel-forming mucins, which form into a biopolymer network with low viscous and elastic moduli allowing for efficient transport at airway surfaces.^1^ The mucus layer is continually transported by the coordinated beating of cilia on airway epithelial cells, which is the primary mechanism of removing mucus from the lungs, referred to as mucociliary clearance (MCC).^2^ The network structure of the mucus gel enables the trapping and removal of micro- and nanoscale particles via MCC. MCC provides our body's first line of defense against inhaled pathogens such as respiratory viruses.^3^ Similarly, MCC may also limit the bioavailability of therapeutic nanocarriers, posing a significant challenge in inhaled drug delivery applications.^4^

Whether therapeutics or pathogens, the ability of particles to overcome the mucus barrier is dependent on the particle's surface chemistry, size, shape, and rigidity. Particles with a positively charged or hydrophobic surface adhere strongly to the mucus network due to their net negatively charged and hydrophobic domains within mucins.^1^ It has been shown in previous work that mucoadhesion can be limited by coating hydrophobic and/or charged nanoparticles (NPs) in a layer of polyethylene glycol (PEG).^5^ PEG is a hydrophilic, net-neutral polymer, which prevents hydrophobic and charge-mediated interactions with mucins, allowing particles to move more freely through the mucus layer. A previous study has also shown that nanoparticles with peptide coatings having a net-neutrally charged amino acid sequence are capable of rapid diffusion through the mucus network.^6^ For pathogens like influenza, specific interactions of viral particles with mucin glycans can potentially lead to their entrapment in the mucus layer.^7^ Depending on their dimensions, nanoscale particles may also be physically immobilized within the mucus gel when much larger than the mucus pore size which ranges from 100 to 500 nm.^8^ Particles that are smaller than the mesh network are likely to diffuse at a rate that corresponds to the viscosity of the fluid-filled pores between the network fibers.^9^ However, heterogeneity of the mucus network can also lead to non-uniformity in nanoparticle diffusion with more rapid movement in regions with decreased mucin density, whereas regions with increased mucin density can significantly hinder and immobilize particles even when densely coated in PEG.^2^

To characterize the diffusion and transport of nanomaterials, multiple particle tracking (MPT) analysis is a commonly used technique to directly measure the diffusion rate of individual nanoparticles within mucus and other complex biological fluids.^10^ However, as a result of the heterogeneity in the mucus gel previously noted, the type of diffusion may vary substantially as of function of spatial position. This complicates interpretation of MPT experiments as the ability of nanoscale particles to navigate through the mucus barrier will be highly dependent on the mode of diffusion. Furthermore, MPT analysis is most often limited to timescales on the order of seconds whereas nanomaterial diffusion and transport through mucus in the lung and other mucosal tissues occur over physiological timespans over minutes to hours.^11^ Thus, new analytical pipelines have been sought to connect MPT analysis to physical models of traversal time through the mucus barrier.^12^ However, these models often require generalized assumptions about the mode of diffusion, which are unlikely to fully capture NP dynamics within the highly complex mucus microenvironment.

Toward this end, we combine machine learning and mathematical models to predict the passage times of nanoparticles and viruses across the mucus barrier. Specifically, we use a previously developed machine learning-based analysis called MotionNet (MoNet) to classify the type of diffusion exhibited in individual particle trajectories.^13^ With these classifications determined, the percentage of particles able to penetrate a mucus layer with a physiological thickness of 10 *μ*m is calculated using diffusion mode-dependent analytical expressions.^14^ We applied this analytical approach to interpret trajectories of PEGylated fluorescent nanoparticles (NP) in human mucus acquired using MPT in 30 distinct human mucus samples. In addition, we evaluated expected passage times for influenza in human mucus using our recently published dataset.^15^ The results of this work provide an approach to predict the timescales, in which synthetic and viral nanoparticles diffuse through the mucus layer in the airway and other mucosal tissues.

## II. RESULTS

### A. Predicted classification of muco-inert nanoparticles in human mucus samples

**FIG. 1.**
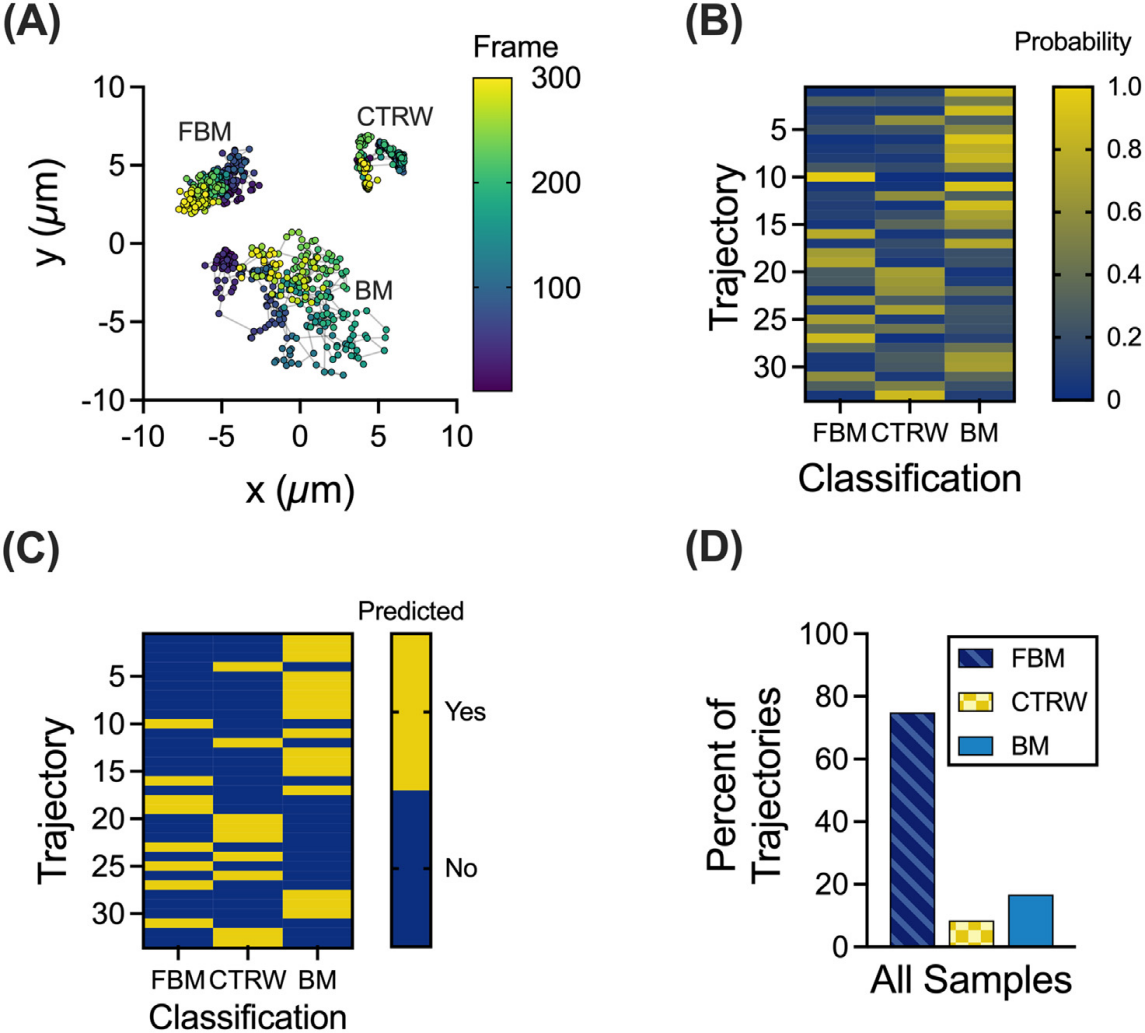
Machine learning based analysis to classify NP trajectories. (a) Representative trajectories for each NP motion type; FBM, BM, and CTRW. Trajectory color corresponds to the frame of video with purple as the first frame and yellow as the last frame. Nanoparticle motion was captured at a frame rate of 33.3 frames per second. (b) and (c) Representative walk-through of MoNet analysis with the probabilities for each trajectory (b) and the predicted motion (c). (d) Percent of trajectories exhibiting FBM, CTRW, or BM across all samples (n = 30 samples).

We utilized the MoNet analysis developed in the previous work^13^ on experimental MPT data of 100 nm NP diffusion within human mucus samples collected from endotracheal tubes (ETs). Using this analysis, we considered three diffusion modes: Brownian motion (BM), fractional Brownian motion (FBM), and continuous time random walk (CTRW). BM is a standard model for diffusion in a Newtonian fluid (e.g., water), where a particle undergoes a random walk, taking steps left or right with equal probability.^14^ BM would most likely be reflective of free diffusion within aqueous regions of the gel. FBM and CTRW are both sub-diffusive models but driven through distinct processes. CTRW motion is characterized by random jumps in time and space leading to “hopping” NP diffusion. In FBM, NPs follow a random walk, but subsequent steps are anti-correlated, meaning that there is a higher probability that the next step will be in the opposite direction than the previous step.^13^ While FBM is more commonly observed in viscoelastic fluids,^16^ hopping motion has been more recently considered as a mode of NP diffusion through biopolymer matrices.^17^ Based on the MoNet analysis, we observed all three forms of diffusion in our dataset with representative trajectories for each type of motion shown in Fig. 1(a). A representative walkthrough of the MoNet analysis is shown in Figs. 1(b) and 1(c) for data collected in an individual patient sample. The MoNet analysis determines the probability that an individual particle will exhibit one of the three types of motion [Fig. 1(b)] and then predicts the type of motion for that individual particle based on these probabilities [Fig. 1(c)]. Using this analysis, we found the predominant type of NP motion in all samples tested to be FBM with a smaller fraction of NPs exhibiting CTRW or BM [Fig. 1(d)].

### B. Classification of trajectories correlates with extent of nanoparticle confinement within mucus network

**FIG. 2.**
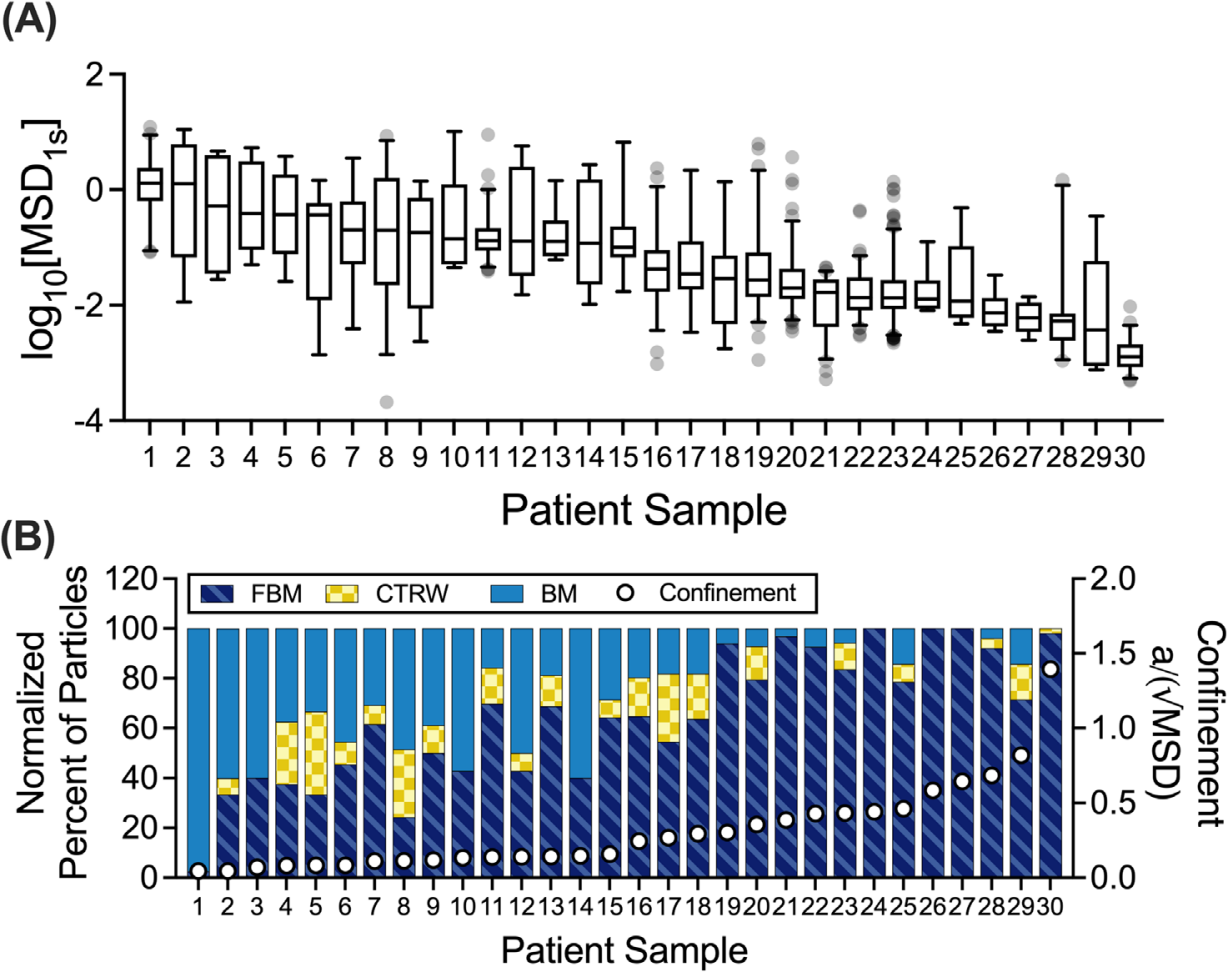
Patient-to-patient variation in NP mobility and diffusion classifications. (a) Distribution of log_10_[MSD_1s_] values for each individual NP in each patient sample (n = 30 samples). Patient samples were ordered by decreasing median log_10_[MSD_1s_] values. (b) Normalized percent of particles exhibiting each type of motion in individual patient samples (left y-axis). For comparison on the right y-axis, a dimensionless parameter is shown that reflects the extent of NP confinement calculated as the ratio of particle radius (a) to square root of the median MSD (√MSD). This dimensionless quantity approaches zero for unconfined, highly diffusive NP (i.e., √MSD ≫ a). The value of this parameter approaches or exceeds 1 for particles confined within the mucus gel network (i.e., when √MSD ∼ a). Whiskers are drawn down to the fifth percentile up to the 95th percentile, and outliers are plotted as points.

Due to inherent patient-to-patient variability in mucus properties,^18–20^ we compared the type of NP diffusion exhibited in individual patient samples. Based on the measured logarithm based 10 (log_10_) of the mean squared displacement (MSD) values at a lag time of 1 s (log_10_[MSD_1s_]), a measure of diffusivity [Fig. 2(a)], we found that the diffusivity varied dramatically from sample to sample with median log_10_[MSD_1s_] spanning over ∼3 orders of magnitude. We utilized the MoNet analysis on the individual trajectories of each sample and compared the percent of NPs exhibiting each type of motion. We then observed how the frequency of diffusion modes changed in individual samples as a function of the NP confinement within the mucus network [Fig. 2(b)]. The extent of NP confinement was estimated based on a dimensionless parameter calculated as the ratio of the probe radius (a) to the square root of the median MSD (MSD^1/2^). Interestingly, we observed that as the NP became more confined (with a/MSD^1/2^ approaching or exceeding 1)—the percent of FBM NPs increased and the percent of BM NPs decreased. CTRW diffusion was observed least frequently in individual samples (0%–33.3%) but was observed in 20 out of 30 samples tested.

### C. Prediction of anomalous diffusion exponents and diffusion coefficients

**FIG. 3.**
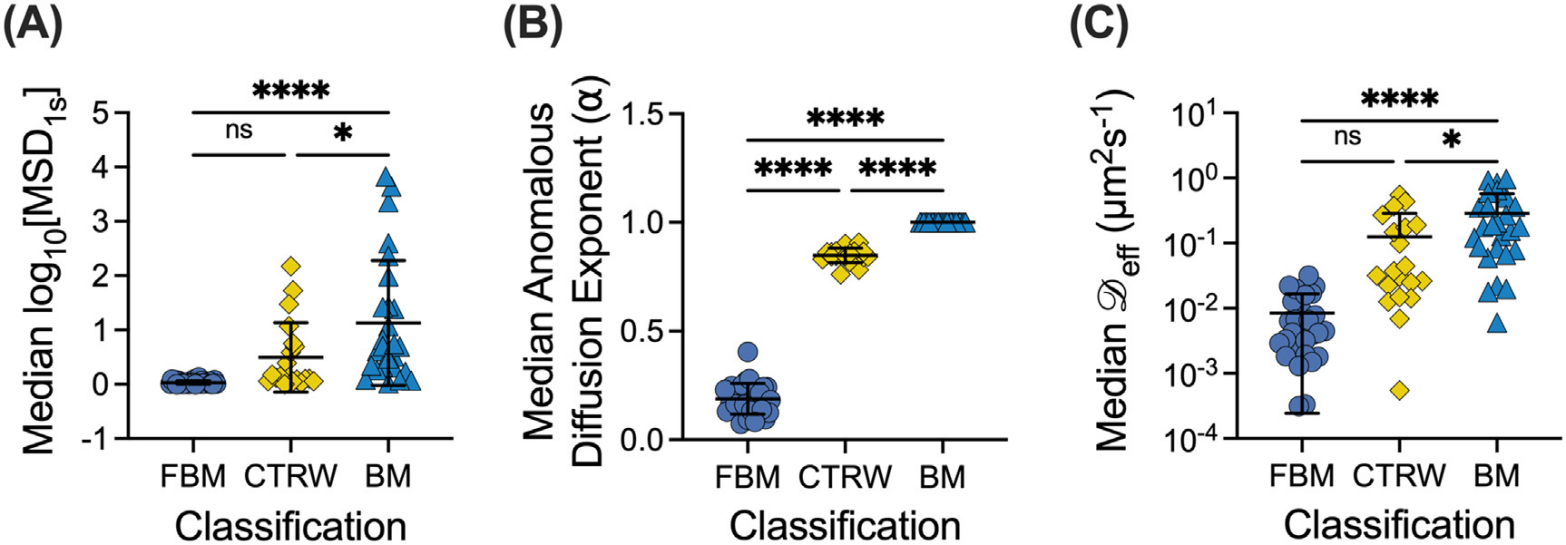
NP mobility in human mucus is highly dependent on the mode of diffusion. (a) Median log_10_[MSD_1s_] values for NP exhibiting each type of motion across all patient samples (n = 30 samples). (b) and (c) Median predicted anomalous diffusion exponents (b) and median diffusion coefficients (c) for particles exhibiting each type of motion across all patient samples. Line at mean and error bars represent standard deviation. Dataset statistically analyzed with one-way analysis of variance (ANOVA) and Šídák's multiple comparisons test: ^*^p <0.05, ^**^p <0.01, ^***^p <0.001, ^****^p <0.0001.

To further investigate the correlation between the diffusion rate of NP in mucus and the classification of motion, we compared log_10_[MSD_1s_] values in each classification (BM, FBM, CTRW) across all samples tested [Fig. 3(a)]. We observed significant differences in the log_10_[MSD_1s_] values, where as expected FBM NPs have the lowest diffusivity, BM NPs have the highest diffusivity, and diffusivity of CTRW NPs was in an intermediate range (i.e., FBM < CTRW < BM). Using the MoNet analysis, we generated the predicted anomalous diffusion exponent (α) for each NP [Fig. 3(b)], which was, subsequently, used to calculate the effective diffusion coefficient ( D_eff_) for each NP [Fig. 3(c)]. We note α = 1 for all BM NPs indicative of normal diffusion and α < 1 for FBM or CTRW NPs indicative of anomalous sub-diffusion. Based on the measured MSD and α, we find effective diffusion coefficients vary significantly between diffusion types following the same trend as observed in the log_10_[MSD_1s_] values; FBM NPs have the lowest effective diffusion coefficients, BM NPs have the highest, and the CTRW NPs are in an intermediate range.

### D. Probability of muco-inert nanoparticles traversing through the mucus barrier

**FIG. 4.**
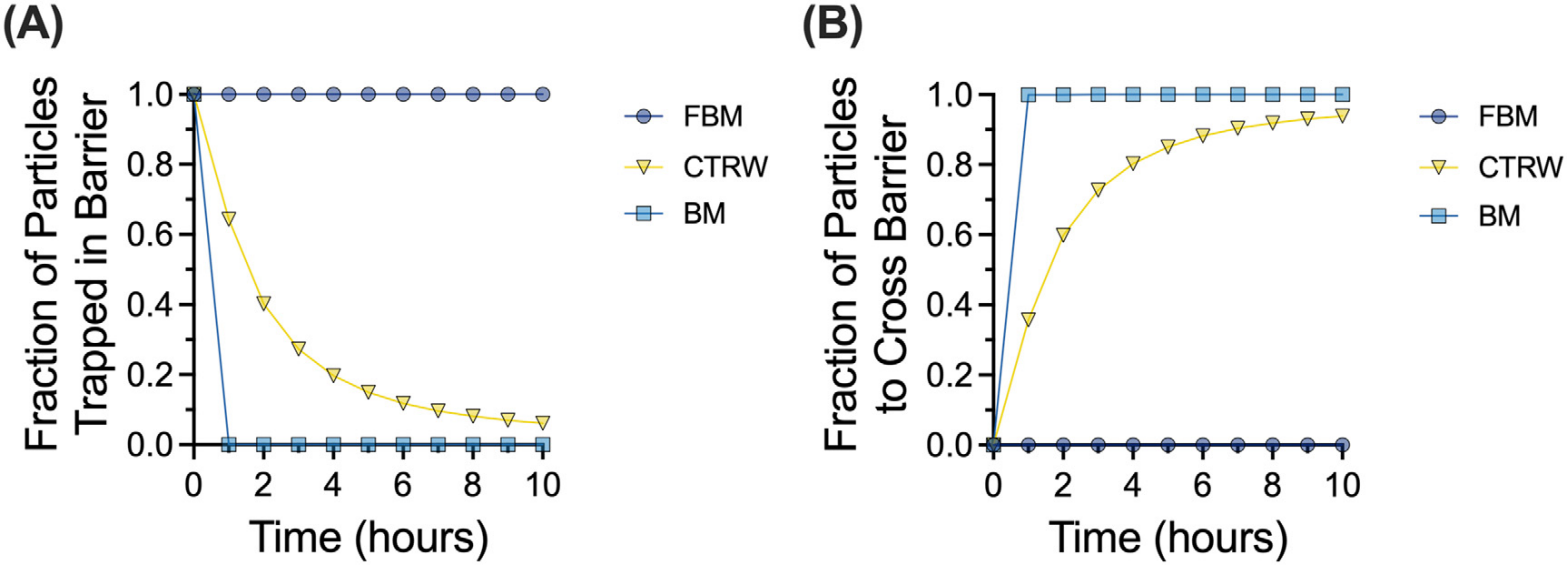
Probability of NPs crossing the mucus barrier based on the MoNet analysis. (a) Survival function showing fraction of particles trapped in the mucus barrier layer of 10 *μ*m thickness over time. (b) Cumulative distribution function showing the fraction of particles that cross the 10 *μ*m mucus barrier over time.

Reframing these data in a physiological context, we utilized a mathematical model developed in the previous work^14^ to predict mean traversal times across the mucus layer. The median anomalous diffusion exponents and diffusion coefficients for each type of motion (Table I) were used to generate the survival function [Fig. 4(a)], indicating which fraction of particles would remain trapped in a 10 *μ*m mucus layer over time. Taking the inverse of the survival function, we obtain the cumulative distribution function [Fig. 4(b)], indicating the fraction of particles would cross through the mucus layer over time. Of note, we predict none of the FBM particles would travel across the mucus layer within a 10-h timespan. We predict all BM NPs would reach the underlying cells in ∼30 min, whereas 30%–40% of CTRW NPs would bypass the mucus layer in the 30–60-min timeframe.

**TABLE I.** Median α and D_eff_ values across all samples.

	Anomalous diffusion exponent (α)	Diffusion coefficient ( D_eff_, *μ*m^2^ s^−1^)
FBM	0.1878	0.003 568
CTRW	0.8541	0.026 11
BM	1	0.192 5

### E. Variation of percent solids in human mucus effects' particle diffusion time

**FIG. 5.**
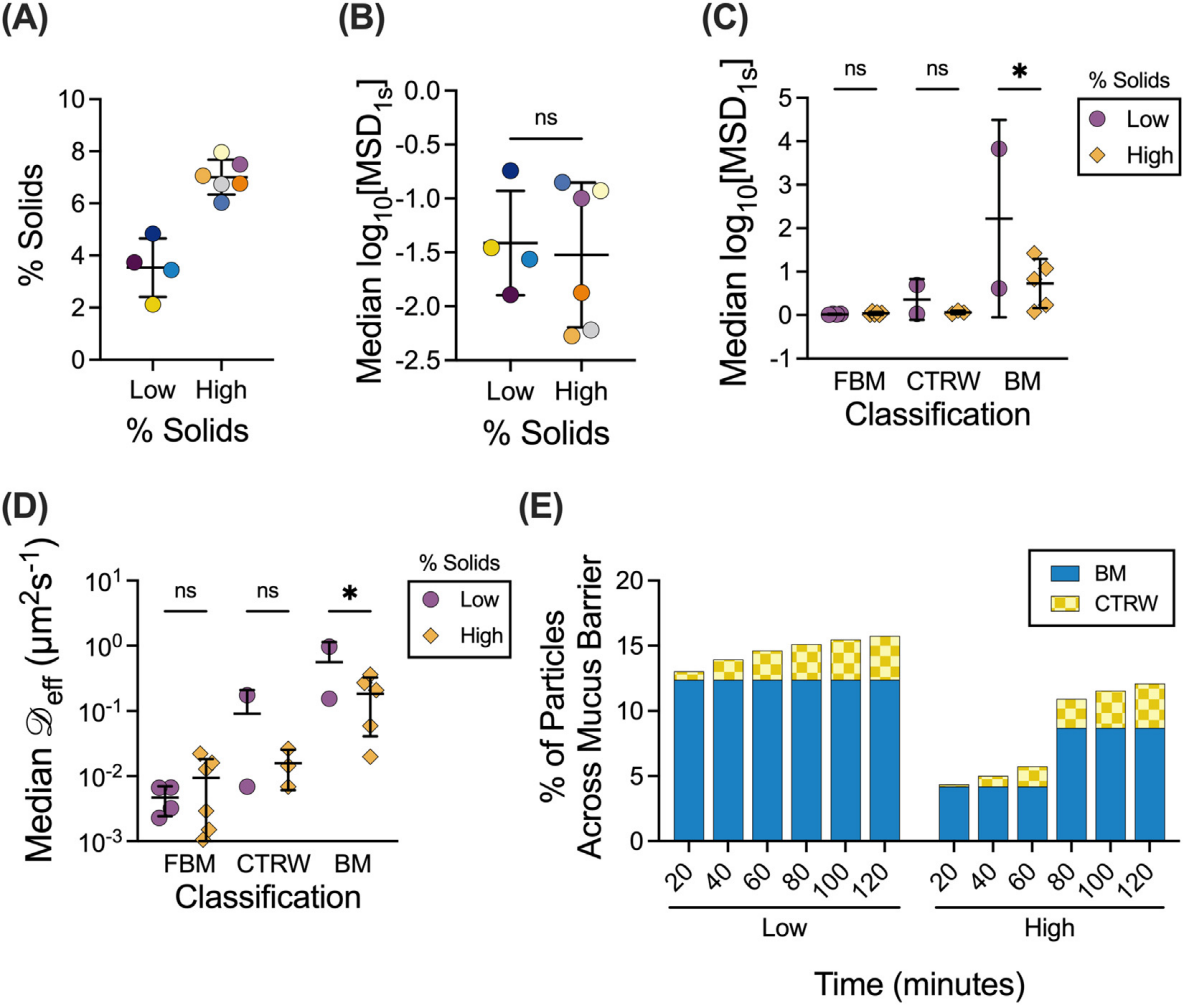
Effect of the solids concentration on NP mobility and penetration through the mucus barrier. (a) Human mucus samples (n = 10 samples total) classified into low percent solids (n = 4 samples) or high percent solids (n = 6 samples). Lines indicate mean and standard deviation. (b) and (c) Distribution of log_10_[MSD_1s_] values for NPs in patient samples classified as having low or high percent solids (b) and NPs of classified by motion type and percent solids (c). (d) Distribution of effective diffusion coefficients ( D_eff_) for particles exhibiting each type of motion. (e) Percent of particles to cross the 10 *μ*m mucus barrier over time, calculated from the cumulative distribution function and normalized percent of particles. Line at mean and error bars represents standard deviation. Dataset statistically analyzed with one-way ANOVA and Šídák's multiple comparisons test: ^*^p <0.05, ^**^p <0.01, ^***^p <0.001, ^****^p <0.0001.

The mucus barrier may also be altered as a function of the overall solids concentration, where more concentrated mucus will likely present a more significant barrier to effective nanoparticle drug delivery.^1^ To account for the potential changes to the mucus barrier because of varied solids concentrations, ten samples were grouped based on measured percent solids [Fig. 5(a)] with 5% solids as the transition point between low and high percent solids. The log_10_[MSD_1s_] values are grouped by percent solids [Fig. 5(b)] and further separated by each type of motion [Fig. 5(c)]. The α and D_eff_ values for each type of motion were calculated for each sample and grouped by the percent solids. The distribution of D_eff_ values for NPs exhibiting each type of motion is shown for samples grouped by low and high percent solids [Fig. 5(d)]. Notably, there is a significant difference between the D_eff_ values for BM NPs with BM NPs in high percent solids samples having lower D_eff_ values. The median α and D_eff_ values for low and high percent solids (Table II) were then used in the particle survival analysis. The resulting cumulative distribution function was used with the normalized percentage of particles for each type of motion to calculate the percentage of particles that would be able to cross the 10 *μ*m mucus barrier [Fig. 5(e)]. The resulting percentages indicated that in mucus with low percent solids, a larger fraction of BM and CTRW particles are predicted to cross the mucus barrier in a shorter amount of time than in mucus with high percent solids. However, regardless of percent solids, the FBM NPs are not predicted to cross the mucus barrier.

**TABLE II.** Median α and D_eff_ values across samples grouped by percent solids.

	Anomalous diffusion exponent (α)		Diffusion coefficient ( D_eff_, *μ*m^2^ s^−1^)	
	Low % solids	High % solids	Low % solids	High % solids
FBM	0.2173	0.174	0.004 806	0.002 777
CTRW	0.8614	0.8641	0.032 42	0.014 35
BM	1	1	0.293 1	0.060 79

### F. Probability of influenza A virus particles crossing the mucus barrier

**FIG. 6.**
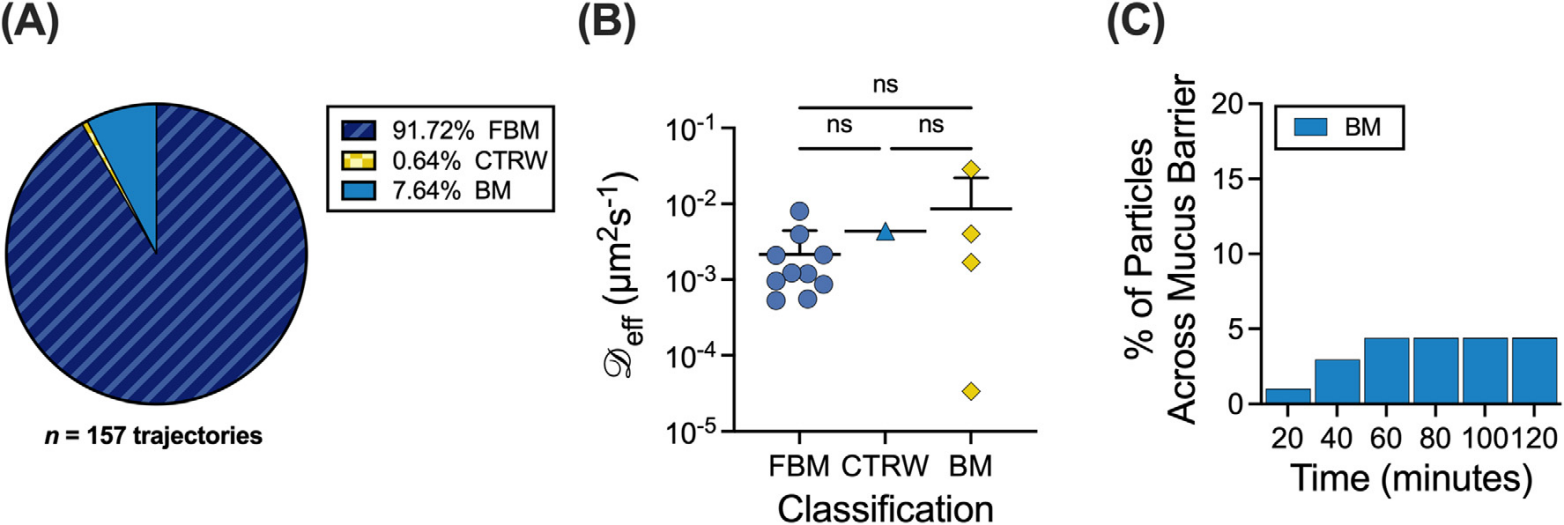
Application to influenza A virus diffusion in human mucus. (a)–(c) MoNet analysis applied to experimental data from Ref. 15. (a) Percent of IAV particles exhibiting each type of motion (n = 10 samples). (b) Median effective diffusion coefficients ( D_eff_) for IAV in human mucus classified by motion type. (c) Percent of IAV particles to cross the 10 *μ*m mucus barrier over time, calculated from the cumulative distribution function and normalized percent of particles. Lines at mean and error bars represent standard deviation. Dataset statistically analyzed with one-way ANOVA and Šídák's multiple comparisons test: ^*^p <0.05, ^**^p <0.01, ^***^p <0.001, ^****^p <0.0001.

We then applied the MoNet and particle survival analyses to a recently published dataset for influenza A virus (IAV) particle diffusion in human mucus from ten different patient donors.^15^ The trajectories were classified via the MoNet analysis and the percent of the IAV particles exhibiting each type of motion are shown in Fig. 6(a). The distribution of D_eff_ values for the IAV is grouped by motion type and is shown in Fig. 6(b). The median α and D_eff_ values for the IAV particles (Table III) were then used in the particle survival analysis. The percentage of particles that would be able to cross the 10 *μ*m mucus barrier based on the cumulative distribution function and the normalized percent of particles is shown in Fig. 6(c). We found only a small percentage of IAV particles are predicted to cross the mucus barrier, all of which are classified as BM particles.

**TABLE III.** Median α and D_eff_ values for IAV in human mucus.

	Anomalous diffusion exponent (α)	Diffusion coefficient ( D_eff_, *μ*m^2^ s^−1^)
FBM	0.097 14	0.001 23
CTRW	0.857 6	0.004 367
BM	1	0.012 35

## III. DISCUSSION

In this work, we examined the different types of particle motion that NPs exhibited in human airway mucus samples collected and analyzed *ex vivo*. We showed that FBM is the predominant type of motion (Fig. 1) and identified a potential link between the network structure, quantified by extent of NP confinement, and the distribution of diffusion modes observed (Fig. 2). We found clear differences in diffusivity for each type of particle based on measured log_10_[MSD_1s_] and effective diffusion coefficients (Fig. 3). As expected, BM NPs traverse the mucus layer most rapidly, whereas CTRW NPs can travel through the mucus layer given adequate time (Fig. 4). However, FBM NPs remain trapped in the mucus layer even at longer timescales up to 3 weeks (data not shown). This suggests that for effective drug delivery, particles would need to exhibit either BM or CTRW movement to traverse the mucus barrier to reach the underlying airway epithelium.

We also found that the percent solids play an important role in how quickly NPs cross the barrier, where a much larger fraction of particles traversed through the mucus barrier with sub-5% total solids content (Fig. 5). This could be explained by the overall density and reduced mucus network pore size expected in mucus with high percent solids (>5%), which more greatly impedes NP movement. This observation is further corroborated by the increased percentage of CTRW NPs and decreased percentage of BM NPs in mucus with high percent solids. However, there was no clear correlation between the percentage of solids in individual samples and the percentage of particles for each classification or the D_eff_ and α values (supplementary material Figs. 1 and 2). When considering rapidly moving BM NPs, we find the median effective diffusion coefficients for BM NPs are ∼fivefold higher at low solids concentration as compared to NPs dispersed in mucus with high percent solids [Fig. 5(d) and Table II]. This is likely due to crowding in mucus with high percent solids as compared to mucus with low percent solids. These findings are consistent with a previous study by Markovetz *et al.*, in which they found that nanoparticles in human mucus exhibited restricted, sub-diffusive motion, and particle motion restriction increased with increased concentration of solids.^20^ However, we note our study did not account for possible dehydration of airway mucus during intubation, which if present may lead to altered physical properties of samples collected.

The ramifications of these results are particularly interesting, as previous studies have shown that the percent solids concentration of airway mucus positively correlates with disease severity in obstructive lung diseases such as cystic fibrosis and chronic bronchitis.^21^ Thus, one would anticipate nanomedicine used in the treatment of these disorders would encounter a mucus barrier with increased percent solids, which could potentially reduce their efficacy However, at very high percent solids, 7%–10%, the cilia compresses, causing reduced MCC,^21^ and this may provide a larger timeframe for NPs to successfully reach the underlying airway epithelial cells. Through these analyses, we can interpret experimental MPT data collected and make more accurate predictions of the NP passage through the mucus barrier in healthy airways and airways of individuals with respiratory disease.

We have also applied these analyses to the diffusion of IAV particles in airway mucus. Intriguingly, while IAV has been shown to be primarily subdiffusive in previous studies,^22,23^ there is a small percentage, 2%–4%, of particles that are predicted to cross the mucus barrier in a physiological 30–60-min time frame, all of which exhibit BM. The outer envelope of IAV contains neuraminidase (NA) and hemagglutinin (HA), which impact their interactions with airway mucus.^24^ While HA binds to sialic acid, which is present on mucins, NA is responsible for cleaving sialic acid. The resulting NA-driven movement is likely responsible for the small percentage of particles predicted to cross the mucus barrier.^24^ The remaining particles that are effectively trapped and exhibiting primarily FBM are likely due to steric and adhesive interactions with the mucus network that NA activity alone is not able to overcome.^15^ Interestingly, there were no significant differences in the anomalous diffusion exponent (α), diffusion coefficient ( D_eff_), and percentage of particles in each classification for IAV and NPs (supplementary material Fig. 3). This would further indicate that steric interactions have a stronger role than adhesive interactions in restricting the IAV passage through the mucus barrier. Importantly, our results demonstrate how the diffusion of respiratory viruses in mucus can be analyzed using these methods, allowing for further understanding of infectious disease pathobiology.

In summary, we utilized machine learning and mathematical models to further interpret multiple particle tracking of NP diffusion through the mucus barrier. Comparable to previous reports, we found most NPs in mucus exhibit sub-diffusive motion with the majority exhibiting FBM and these particles are unlikely to reach the airway surface in a physiological timespan. Our results suggest NPs exhibiting BM are the primary population expected to penetrate the mucus barrier prior to clearance. However, hopping CTRW NPs can navigate through the mucus barrier albeit over prolonged time frames. Our results suggest that the fate of a 100 nm nanoparticle in the mucus barrier will strongly depend on the percentage of solids and network pore size, which subsequently impacts the diffusion time through the mucus layer. This study establishes a workflow to rigorously evaluate the mucus-penetrating capabilities of NPs used in drug delivery applications. We have also shown these analyses can be used in studies of viral trafficking through the mucus barrier to complement standard assays used in infectious disease research.

## IV. METHODS

### A. Nanoparticle preparation

As previously reported, carboxylate modified fluorescent polystyrene nanoparticles (NP; Life Technologies) with a diameter of 100 nm were coated with 5-kDa methoxy polyethylene glycol (PEG)-amine (Creative PEGWorks) via a carboxyl-amine linkage.^18^ The particle size distribution and surface charge were confirmed via dynamic light scattering using the NanoBrook Omni (Brookhaven Instruments). We confirmed the presence of a dense PEG coating on NPs based on the measured zeta potential of 0.04 ± 0.71 mV.

### B. Human mucus collection

Human mucus was collected under an IRB-approved protocol at the University of Maryland Medical Center (UMMC; Protocol No. HP-00080047). Samples were collected by the endotracheal tube (ET) method, as previously described.^18^ ETs were collected from 30 donors after intubation as a part of general anesthesia at UMMC. The data presented here are from six male and nine female subjects with a mean age of 57 ± 13 years. (Note: demographic data are not available for 15 patients.) All the available demographics information for samples used in this work is included in supplementary material Table 1 and supplementary material Fig. 4. To collect mucus from the ET, the last approximately 10 cm of the tubes were cut, including the balloon cuff, and placed in a 50 ml centrifuge tube. The ET tube was suspended in the tube with a syringe needle and was then spun at 220 g for 30 s, yielding 100–300 *μ*l of mucus. Mucus with visible blood contamination was not included in the analysis. Samples were stored in 4 °C immediately after collection and imaged within 24 h of collection.

### C. Percent solids analysis

To determine the percent solids, 100–150 *μ*l of the human mucus sample was placed on the pre-weighed weigh paper, and the total mass was measured. The sample was then dried on a hotplate for at least 2 h or until there was no weight change. The dried sample was weighed, and the percent solids was calculated as the difference between the wet and dried sample weight. Due to collected volume, percent solids were measured for ten of the 30 human mucus samples.

### D. Fluorescence video microscopy

Samples were prepared for imaging by placing a vacuum grease coated O-ring on microscope cover glass. The sample was then applied to the center of the well and sealed with a coverslip. For each sample, 1 *μ*l of PEG-coated NPs were added to 20 *μ*l human mucus (approximately 2 × 10^6^ particles/sample) in the center of the slide well and stirred with a pipette tip prior to imaging. Samples were then equilibrated for 30 min at room temperature prior to imaging. Slides were imaged using a Zeiss LSM 800 inverted microscope with a 63× water-immersion objective. Multiple 10 s videos were recorded at 33.3 frames per second for each sample. Similar methods were used for measuring influenza A virus diffusion in human mucus and are described in detail in our previously published work.^15^

### E. Multiple particle tracking (MPT) analysis

Acquired fluorescence microscopy videos were processed using a previously developed MATLAB (The MathWorks, Natick, MA) based analysis code to isolate and track imaged particles.^10,25,26^ For each video, the mean squared displacement (MSD) was calculated as $\left\langle \text{MSD}\left(\tau \right)\right\rangle =\left\langle \left(x^{2}+y^{2}\right)\right\rangle$ for each particle. Due to the nature of MPT, NPs were tracked for a maximum of 10 s due to their motion out of the focal plane. To minimize the dynamic and static error in our measurements,^26^ a lag time of 1 s was used as a representative value for comparison between conditions. The 100 nm nanoparticles have a median static error MSD of 4.39 × 10^−5^ *μ*m^2^/s at 1 s. The estimated confinement of the NP was calculated as $a/\sqrt{{\text{MSD}}_{1\;s}}$, where MSD_1 s_ is the measured MSD at τ = 1 s and *a* is the NP radius. The same methods were used in the analysis of influenza A virus diffusion in human mucus as discussed in our previously published work.^15,27^

### F. Machine learning analysis

A previously developed convolutional neural network model referred to as MotionNet (MoNet) was used to determine the probability of a single trajectory of length 300 frames will fall into the diffusion category of Brownian motion (BM), fractional Brownian motion (FBM), or continuous time random walk (CTRW).^13^ Once classified by the type of diffusion, MoNet predicts anomalous diffusion exponents (α) for FBM and CTRW particles.^13^ The effective diffusion coefficient ( D_eff_) was then calculated for each particle as $\left\langle \text{MSD}\left(\tau \right)\right\rangle =4D_{\alpha }\tau^{\alpha }$, where *α* is the anomalous diffusion exponent predicted by MoNet and τ is the lag time.^28^ As some particles move out of frame during imaging, trajectories acquired from the MPT analysis were filtered in MATLAB (The MathWorks, Natick, MA) to isolate trajectories that were tracked for 300 frames. On average, 34 NP trajectories were kept for MoNet analysis per sample tested. In rare cases, a minimum of 5 NP trajectories were kept.

### G. Particle survival analysis

A previously developed mathematical model is used for predicting the first traversal times for nanoparticles exhibiting diffusive (Brownian) motion and fractional sub-diffusive motion (i.e., CTRW and FBM).^14^ The survival function for diffusive motion (*S*_D_) is given as

$$S_{D}\left(t\right)=\frac{4}{\pi }\sum_{n=0}^{\infty }\left[\;\text{exp}\;\left(-{\left(\frac{\left(2n+1\right)\pi }{2h}\right)}^{2}Dt^{\alpha }\right)\frac{{\left(-1\right)}^{n}}{2n+1}\right],$$ (1)

where D is the diffusion coefficient and *h* is the distance to the absorbing boundary. The survival function for sub-diffusive motion (*S*_SD_) is given as

$$S_{\text{SD}}\left(t\right)=\frac{4}{\pi }\sum_{n=0}^{\infty }\left[E_{\alpha }\left(-{\left(\frac{\left(2n+1\right)\pi }{2h}\right)}^{2}D_{\alpha }t^{\alpha }\right)\frac{{\left(-1\right)}^{n}}{2n+1}\right],$$ (2)

where $E_{\alpha }\left(z\right)=\sum_{k=0}^{\infty }\frac{z^{k}}{\Gamma (1+\mathrm{\alpha k})}$ is the Mittag–Leffler function.^14^ The cumulative distribution function (*F*), given as F(t)=1−S(t), gives the fraction of particles that cross the absorbing boundary.^14^ A physiological thickness (*h*) of 10 *μ*m was used for all calculations. The anomalous diffusion exponents (α) and effective diffusion coefficient ( D_eff_) from the MoNet analysis were used to calculate the survival functions for each type of particle using their respective equations. The percentage of particles that would be able to cross the mucus barrier was calculated from the *F* and the normalized percentage of particles for each type of motion using the following equation:

$${\%\;\text{particles}\;\text{to}\;\text{cross}\;\text{mucus}\;\text{barrier}}_{\;}=F\times \text{Normalized}\;\%\;\text{Particles}.$$ (3)

### H. Statistical analysis

Data were statistically analyzed using GraphPad Prism 9 (GraphPad Software, San Diego, CA).

## SUPPLEMENTARY MATERIAL

See the supplementary material for the additional data, including correlation between percent solids and NP diffusion (supplementary material Figs. 1 and 2), comparison of NPs and IAV diffusion (supplementary material Fig. 3), patient demographics for clinical samples used in our study (supplementary material Table 1), and correlation between age or sex and NP diffusion (supplementary material Fig. 4).

## Data Availability

The data that support the findings of this study are available from the corresponding author upon reasonable request.

## References

[1] D. Song , D. Cahn , and G. A. Duncan , Langmuir 36, 12773 (2020). 10.1021/acs.langmuir.0c02410 33094612

[2] B. Huck , X. Murgia , S. Frisch , M. Hittinger , A. Hidalgo , B. Loretz , and C.-M. Lehr , Adv. Drug Delivery Rev. 183, 114141 (2022). 10.1016/j.addr.2022.114141 35149123

[3] J. V. Fahy and B. F. Dickey , N. Engl. J. Med. 363, 2233 (2010). 10.1056/NEJMra0910061 21121836 PMC4048736

[4] M. Ernst , T. John , M. Guenther , C. Wagner , U. F. Schaefer , and C.-M. Lehr , Biophys. J. 112, 172 (2017). 10.1016/j.bpj.2016.11.900 28076809 PMC5233549

[5] C. S. Schneider , Q. Xu , N. J. Boylan , J. Chisholm , B. C. Tang , B. S. Schuster , A. Henning , L. M. Ensign , E. Lee , and P. Adstamongkonkul , Sci. Adv. 3, e1601556 (2017). 10.1126/sciadv.1601556 28435870 PMC5381952

[6] L. D. Li , T. Crouzier , A. Sarkar , L. Dunphy , J. Han , and K. Ribbeck , Biophys. J. 105, 1357 (2013). 10.1016/j.bpj.2013.07.050 24047986 PMC3785869

[7] E. Iverson , L. Kaler , E. L. Agostino , D. Song , G. A. Duncan , and M. A. Scull , Viruses 12, 1425 (2020). 10.3390/v12121425 PMC7763686 33322395

[8] J. Witten and K. Ribbeck , Nanoscale 9, 8080 (2017). 10.1039/C6NR09736G 28580973 PMC5841163

[9] J. Witten , T. Samad , and K. Ribbeck , Curr. Opin. Biotechnol. 52, 124 (2018). 10.1016/j.copbio.2018.03.010 29674157 PMC7132988

[10] B. S. Schuster , L. M. Ensign , D. B. Allan , J. S. Suk , and J. Hanes , Adv. Drug Delivery Rev. 91, 70 (2015). 10.1016/j.addr.2015.03.017 PMC4813524 25858664

[11] G. A. Duncan , J. Jung , J. Hanes , and J. S. Suk , Mol. Ther. 24, 2043 (2016). 10.1038/mt.2016.182 27646604 PMC5167788

[12] J. M. Newby , I. Seim , M. Lysy , Y. Ling , J. Huckaby , S. K. Lai , and M. G. Forest , Adv. Drug Delivery Rev. 124, 64 (2018). 10.1016/j.addr.2017.12.002 PMC5809312 29246855

[13] V. Jamali , C. Hargus , A. Ben-Moshe , A. Aghazadeh , H. D. Ha , K. K. Mandadapu , and A. P. Alivisatos , Proc. Natl. Acad. Sci. 118, e2017616118 (2021). 10.1073/pnas.2017616118 33658362 PMC7958372

[14] A. M. Erickson , B. I. Henry , J. M. Murray , P. J. Klasse , and C. N. Angstmann , Biophys. J. 109, 164 (2015). 10.1016/j.bpj.2015.05.034 26153713 PMC4572576

[15] L. Kaler , E. Iverson , S. Bader , D. Song , M. A. Scull , and G. A. Duncan , Commun. Biol. 5, 249 (2022). 10.1038/s42003-022-03204-3 35318436 PMC8941132

[16] M. T. Valentine , P. D. Kaplan , D. Thota , J. C. Crocker , T. Gisler , R. K. Prud'homme , M. Beck , and D. A. Weitz , Phys. Rev. E 64, 061506 (2001). 10.1103/PhysRevE.64.061506 11736190

[17] L.-H. Cai , S. Panyukov , and M. Rubinstein , Macromolecules 48, 847 (2015). 10.1021/ma501608x 25691803 PMC4325603

[18] G. A. Duncan , J. Jung , A. Joseph , A. L. Thaxton , N. E. West , M. P. Boyle , J. Hanes , and J. S. Suk , JCI Insight 1, e88198 (2016). 10.1172/jci.insight.88198 27812540 PMC5085601

[19] K. Joyner and G. A. Duncan , Am. J. Physiol. 317, L496 (2019). 10.1152/ajplung.00362.2019 PMC6842912 31508979

[20] M. R. Markovetz , D. B. Subramani , W. J. Kissner , C. B. Morrison , I. C. Garbarine , A. Ghio , K. A. Ramsey , H. Arora , P. Kumar , D. B. Nix , T. Kumagai , T. M. Krunkosky , D. C. Krause , G. Radicioni , N. E. Alexis , M. Kesimer , M. Tiemeyer , R. C. Boucher , C. Ehre , and D. B. Hill , Am. J. Physiol. 317, L498 (2019). 10.1152/ajplung.00238.2019 PMC6842913 31389736

[21] R. C. Boucher , N. Engl. J. Med. 380, 1941 (2019). 10.1056/NEJMra1813799 31091375

[22] X. Yang , L. Steukers , K. Forier , R. Xiong , K. Braeckmans , K. V. Reeth , and H. Nauwynck , PLoS One 9, e110026 (2014). 10.1371/journal.pone.0110026 25333824 PMC4198190

[23] Y.-Y. Wang , D. Harit , D. B. Subramani , H. Arora , P. A. Kumar , and S. K. Lai , Eur. Respir. J. 49, 1601709 (2017). 10.1183/13993003.01709-2016 28122865 PMC6533903

[24] E. de Vries , W. Du , H. Guo , and C. A. M. de Haan , Trends Microbiol. 28, 57 (2019). 10.1016/j.tim.2019.08.010 31629602 PMC7172302

[25] J. C. Crocker and D. G. Grier , J. Colloid Interface Sci. 179, 298 (1996). 10.1006/jcis.1996.0217

[26] K. Joyner , S. Yang , and G. A. Duncan , APL Bioeng. 4, 041508 (2020). 10.1063/5.0013707 33415310 PMC7775114

[27] D. Song , E. Iverson , L. Kaler , S. Bader , M. A. Scull , and G. A. Duncan , ACS Biomater. Sci. Eng. 7, 2723 (2021). 10.1021/acsbiomaterials.0c01728 33871978 PMC8803127

[28] V. Tejedor , O. Bénichou , R. Voituriez , R. Jungmann , F. Simmel , C. Selhuber-Unkel , L. B. Oddershede , and R. Metzler , Biophys. J. 98, 1364 (2010). 10.1016/j.bpj.2009.12.4282 20371337 PMC2849086

